# CCL18 promotes the metastasis of squamous cell carcinoma of the head and neck through MTDH‐NF‐κB signalling pathway

**DOI:** 10.1111/jcmm.14168

**Published:** 2019-02-15

**Authors:** Yuexiang Qin, Juncheng Wang, Gangcai Zhu, Guo Li, Haolei Tan, Changhan Chen, Leiming Pi, Li She, Xiyu Chen, Ming Wei, Zhexuan Li, Zhifeng Liu, Donghai Huang, Yong Liu, Xin Zhang

**Affiliations:** ^1^ Department of Otolaryngology Head and Neck Surgery Xiangya Hospital, Central South University Changsha, Hunan People’s Republic of China; ^2^ Otolaryngology Major Disease Research Key Laboratory of Hunan Province Changsha, Hunan People’s Republic of China; ^3^ Department of Health Management The Third Xiangya Hospital, Central South University Changsha, Hunan People’s Republic of China; ^4^ Department of Otolaryngology Head and Neck Surgery The Second Xiangya Hospital, Central South University Changsha, Hunan People’s Republic of China; ^5^ Department of Head and Neck Surgery, Hunan Cancer Hospital, The Affiliated Tumor Hospital of Xiangya Medical School Central South University Changsha, Hunan People’s Republic of China; ^6^ Department of Otolaryngology Head and Neck Surgery Zhuzhou Central Hospital Zhuzhou, Hunan People’s Republic of China

**Keywords:** CCL18, EMT, metastasis, MTDH, SCCHN

## Abstract

Metastasis is one of the primary causes for high mortality in patients with squamous cell carcinoma of the head and neck (SCCHN). Our previous study showed that chemokine (C‐C motif) ligand 18 (CCL18), derived from tumour‐associated macrophages (TAMs), regulates SCCHN metastasis by promoting epithelial‐mesenchymal transition (EMT) and preserving stemness. However, the underlying mechanism needs to be further investigation. Interestingly, metadherin (MTDH) expression was induced when SCCHN cells were stimulated with recombinant CCL18 protein in this study. Suppressing MTDH expression reversed CCL18‐induced migration, invasion and EMT in SCCHN cells. Furthermore, the NF‐κB signalling pathway was involved in the MTDH knock‐down cells with CCL18 stimulation. We performed ELISA to evaluate the CCL18 levels in the serums of 132 treatment‐naive SCCHN patients, 25 patients with precancerous lesion and 32 healthy donors. Our results demonstrated that serum CCL18 levels were significantly higher in SCCHN patients than patients with precancerous lesion and healthy individuals. CCL18 levels were found to be significantly correlated with tumour classification, clinical stage, lymph node metastasis and histological grade in SCCHN patients. Thus, our findings suggest that CCL18 may serve as a potential biomarker for diagnosis of SCCHN and promote SCCHN invasion, migration and EMT by MTDH‐NF‐κB signalling pathway.

## 1 BACKGROUND

Squamous cell carcinoma of the head and neck (SCCHN) is the sixth most aggressive malignancy worldwide.^1^ Although effective multidisciplinary treatments, including surgery, radiotherapy and chemotherapy, have been applied in clinical practice for decades, the prognosis of SCCHN patients remains poor.^2^ Although metastasis is the primary cause of death in SCCHN patients, there is no explanation for its causal role.^3^, ^4^, ^5^ Elucidation of the underlying mechanism might help generate an effective surveillance of tumour progression and improve the survival outcome of SCCHN patients.

The tumour microenvironment, which consists of a variety of nonmalignant stromal cells, such as tumour‐associated macrophages (TAMs), tumour‐associated dendritic cells and tumour‐infiltrating T cells, is an indispensable participant in tumour progression and metastasis.^6^, ^7^ TAMs, which produce chemokines, are the key players in creating the tumour microenvironment, and they are the most notable migratory hematopoietic cell type.^8^, ^9^, ^10^, ^11^ Chemokine (C‐C motif) ligand 18 (CCL18), also known as pulmonary and activation‐regulated chemokine (PARC), dendritic cell‐derived CC chemokine‐1 (DC‐CK1), alternative macrophage activation‐associated CC chemokine‐1 and macrophage inflammatory protein‐4, has been reported to attract naive T cells or the mantle‐zone B cells, and it primarily targets lymphocytes and immature dendritic cells.^12^ CCL18, a member of the CC chemokine family, is mainly secreted by M2‐type TAMs.^7^, ^8^ Excessive secretion of CCL18 has been observed in multiple human malignancies, including breast carcinoma,^9^, ^10^, ^11^, ^13^ hepatocellular carcinoma,^14^ ovarian cancer,^15^, ^16^ nasopharyngeal carcinoma,^17^ lung cancer,^18^, ^19^, ^20^ colon cancer^21^, ^22^ and pancreatic ductal adenocarcinoma.^23^, ^24^ Expression of CCL18 is associated with tumour initiation and progression, and therefore, could be used to predict the prognosis of patients with breast cancer.^9^ Previously, we demonstrated that CCL18 derived from M2 TAMs promoted the migration and invasion of SCCHN cells through the induction of epithelial‐mesenchymal transition (EMT) and cancer stemness.^25^ However, the molecular mechanism by which CCL18 induced metastasis and EMT in SCCHN cells remains unknown.

Metadherin (MTDH), also known as astrocyte‐elevated gene‐1 and LYsine‐RIch CEACAM1 co‐isolated protein, was initially cloned as HIV‐1 and tumour necrosis factor α‐inducible gene in primary human foetal astrocytes.^26^, ^27^, ^28^, ^29^ MTDH is a downstream target of Ha‐ras and c‐myc and helps in mediating their biological effects, suggesting a potential role of MTDH in tumour initiation and progression.^30^, ^31^ Overexpression of MTDH in normal immortal cloned rat embryo fibroblasts was found to induce anchorage‐independent growth in soft agar and enhance invasion in nude mice.^32^ Several studies have demonstrated that elevated MTDH expression is closely related to poor prognosis of various types of cancers, including breast cancer,^33^ oesophageal squamous cell carcinoma,^34^ gastric cancer,^35^ hepatocellular carcinoma^36^ and non‐small cell lung cancer.^37^ Thus, MTDH is a potential prognostic biomarker for human malignancies. Our previous studies have also confirmed that overexpression of MTDH is highly correlated with lymph node metastasis and shows negative association with poor prognosis in SCCHN patients.^38^, ^39^ MTDH has been demonstrated to be a valuable biomarker for SCCHN progression, and it regulates the metastasis of SCCHN via EMT in vitro.^38^, ^40^

Activation of NF‐κB signalling is associated with a variety of cellular responses, such as inflammation, cell survival, differentiation and proliferation. NF‐κB signalling pathway has also been demonstrated to play an important role in malignant cell mobility.^41^, ^42^, ^43^, ^44^ Accumulated evidence suggests that MTDH mediates the biological effect of the NF‐κB signalling pathway through several mechanisms.^45^ However, it is unknown whether the NF‐κB signalling pathway is involved in the regulation of metastasis by CCL18 in SCCHN cells.

## 2 MATERIALS AND METHODS

### 2.1 Patients and samples

CCL18 levels in fasting serum samples obtained from 132 SCCHN patients, who had undergone surgery between March 2018 and October 2018 at the Department of Otolaryngology in Xiangya Hospital, Department of Otolaryngology in third Xiangya Hospital, Department of Otolaryngology in Hunan Cancer Hospital, Central South University, Changsha, Hunan, China, were assessed. Serum was stored at −80°C for performing ELISA. Clinical data were collected from pathographies of the patients. Preoperative serum was collected from 25 precancerous lesions of SCCHN patients and the negative control group consisted of 32 age‐ and gender‐matched healthy volunteers. Those patients with SCCHN were enrolled in our study who met the following inclusion criteria: no history of radiotherapy or chemotherapy and primary squamous cell carcinoma without previous malignancies. The pathological classification of SCCHN patients was based on UICC (Union for International Cancer Control, 2009 years)‐TNM classification of malignant tumours.^46^ The main clinical and pathological parameters of SCCHN patients are summarized in Table 1. Informed consent was obtained from all the patients before surgery, and all experiments were conducted by following the bioethics rules issued by the Research Ethics Committee of Central South University, Changsha, China.

**Table 1:** Correlations between serum CCL18 levels and clinicopathological parameters in SCCHN patients

Parameters	Number of patients	Serum CCL18 levels			*P*‐value
		Q25	Q50	Q75	
Age (y)					0.811
≤58	67	25780.52	33361.62	49478.32	
>58	65	24936.73	34183.22	51934.74	
Gender					0.630
Female	7	27761.65	41052.69	48769.85	
Male	125	25566.48	33499.44	50344.67	
Smoking history					0.757
No	26	26309.91	34372.16	55895.51	
Yes	106	25316.87	33542.26	49739.36	
Alcohol consumption					0.923
No	76	25813.59	33672.90	49421.03	
Yes	56	25227.24	33884.15	51012.66	
Primary tumour site					0.271
Laryngo	69	25800.02	33361.62	45222.89	
Hypopharynx	21	30540.10	37601.55	66898.76	
Oral cavity	42	23475.27	33858.01	47305.71	
T classification					**0.001**
T1 + T2	65	23728.53	330314.44	41844.56	
T3 + T4	67	29087.33	40558.10	62934.30	
Clinical stage					
I + II	52	22917.32	28755.86	38709.80	**0.000**
III + IV	80	29123.28	40766.82	62605.79	
Lymph node status					**0.001**
N0	73	23467.78	30314.44	42213.84	
N+	*59*	30169.49	40975.54	63401.24	
Histological grade					
G1	52	23467.77	30950.16	41724.13	**0.021**
G2 + G3	80	27257.73	35129.96	59678.64	

CCL18, chemokine (C‐C motif) ligand 18; SCCHN, squamous cell carcinoma of the head and neck.

Bold indicates that *P* is less than 0.05, which is statistically significant.

### 2.2 Cell culture and treatment

Dysplastic oral keratinocyte (DOK), an immortalized non‐malignant cell line, was derived from human oral mucosa. Tu686, an SCCHN cell line derived from human oropharynx carcinoma, was kindly provided by Georgia Chen (Emory University Winship Cancer Institute, Atlanta, USA).^40^ 6‐10B, 5‐8F and CNE2 cell lines, derived from human nasopharyngeal carcinoma (NPC), and FaDU cells, derived from human hypopharynx and larynx carcinoma. All the four cell lines were purchased from the Central Experiment Laboratory of Xiangya Medical School, Central South University, Changsha, China. Monolayer culture of Tu686 cells was maintained in Dulbecco's modified Eagle's medium and Ham's F12 nutrient mixture (1:1, Hyclone, Logan, UT) with 10% foetal bovine serum (FBS) (Gibco, NYC, New York, NY). FaDu cells were cultured in Dulbecco's minimal essential medium (Hyclone) containing 10% FBS. DOK, CNE2, 5‐8F and 6‐10B cells were cultured in RPMI Medium 1640 (Hyclone) containing 10% FBS. Cells were incubated at 37°C in a humidified atmosphere containing 5% CO_2_ and used for experiments when cells were in logarithmic phase. EMT was induced in Tu686 and 6‐10B cells by incubating them with 20 ng/mL recombinant human CCL18 (rhCCL18) protein (Abnova, Taibei, Taiwan), while FaDu cells were incubated with 40 ng/mL of rhCCL18 for 48 hours. These cells were then used for the following experiments. Activation of IκB‐α was inhibited by treating the cells with 5 μmol/L of Bay 11‐7082 (Selleck, Shanghai, China), an specific inhibitor of phosphorylation of IκB‐α, for 48 hours.

### 2.3 Stable transfection

Lentiviral‐MTDH‐shRNA (sc‐77797‐V, Genecopoeia, Santa Cruz, CA), a set of concentrated, transducible viral particles containing three target‐specific constructs encoding 19‐25 nt shRNAs designed to knock down MTDH gene expression in human cells, was introduced into Tu686, 6‐10B and FaDu cells according to the manufacturer's protocol. A control vector containing non‐targeted shRNA was also used to transfect Tu686, 6‐10B and FaDu cells. Forty‐eight hours post transfection, stable cell lines expressing MTDH shRNAs were selected with 5 μg/mL puromycin dihydrochloride for 2 weeks. Transfected cells were expanded and maintained in 3 μg/mL puromycin dihydrochloride and expression of MTDH in these cells was confirmed by Western blot analysis.

### 2.4 Enzyme‐linked immunosorbent assay

CCL18 levels in the serum of SCCHN patients, precancerous lesions of SCCHN patients and healthy volunteers were determined quantitatively using a human PARC (CCL18) ELISA kit (Raybiotech, Atlanta, GA) according to the manufacturer's protocol. Each experiment was performed in triplicate.

### 2.5 Quantitative real‐time PCR

Total RNA was extracted from samples using TRIzol reagent (Life Technologies, Carlsbad, CA) according to the manufacturer's protocol. After cDNA synthesis (All‐in‐One First‐Strand cDNA Synthesis kit, GeneCopoeia Inc, Santa Cruz, CA), quantitative real‐time PCR (qRT‐PCR) was carried out using All‐in‐One qPCR Mix (GeneCopoeia Inc, USA) on ABI 7500HT System (Applied Biosystems, Foster City, CA) using primers described in Table 2. PCR conditions were as follows, 95°C for 10 minutes followed by 40 cycles of 95°C for 10 seconds, 60°C for 20 seconds and 72°C for 27 seconds. The specificity of each qRT‐PCR reaction was verified by melting curve analysis. β‐Actin was used as an internal control. Duplicate reactions were run for each sample and relative change in gene expression was calculated using the 2^−ΔΔCT ^method. All the samples for each experiment were run in duplicate.

**Table 2:** The sequence of primers used for PCR in this study

Gene	Primer sequences (5’–3’)
E‐cadherin	
Forward	TCCATTTCTTGGTCTACGCC
Reverse	CACCTTCAGCCAACCTGTTT
Vimentin	
Forward	TGGCACGTCTTGACCTTGAA
Reverse	GGTCATCGTGATGCTGAGAA
N‐cadherin	
Forward	TGGTGTATGCCGTGAGAAGC
Reverse	TTAAGGTTGGCTTCAGGCTCA
Fibronectin	
Forward	GGCCAGACTCCAATCCAGAG
Reverse	CCGAGCATTGTCATTCAAGG
MTDH	
Forward	ATGGAGGAGGCTGGAATGAA
Reverse	CTCCAGGCAGATGGCTCAGT
Beta actin	
Forward	CTCTTCCAGCCTTCCTTCCT
Reverse	AGCACTGTGTTGGCGTACAG

### 2.6 Western blot analysis

Whole cell protein extracts were collected and Western blot analysis was performed as described previously.^40^, ^47^ Thirty micrograms of total protein was separated on 10% SDS‐PAGE and then transferred to polyvinylidene difluoride membrane (Millipore, Bedford, MA). The membranes were incubated with primary rabbit antibody against PITPNM3 (1:1000 dilution, Genetex, SAN Antonio, TX), E‐cadherin (1:1000 dilution, Santa Cruz, CA), Vimentin and MTDH (1:1000 dilution, Proteintech Group, Chicago, IL), p65 and phosphorylated p65 (1:1000 dilution, Cell Signaling Technology, Danvers, MA) and IκB‐α and phosphorylated IκB‐α (1:1000 dilution, Cell Signaling Technology) at 4°C overnight. After incubation, the membrane was washed with PBST (Phosphate Buffered Saline with Tween‐20; Hyclone, Logan, UT) and incubated with HRP‐labelled goat anti‐rabbit IgG for 1 hour at room temperature. β‐Actin (1:1000 dilution, Proteintech Group) was used as internal control. Each experiment was repeated in triplicates.

### 2.7 Immunofluorescence

Tu686, 6‐10B and FaDu cells were seeded in six‐well plates and incubated for 48 hours. Cells were seeded in 12‐well plates (Corning, Corning, NY) for 1 day, fixed with 4% paraformaldehyde solution for 15 min and permeabilized with 0.3% Triton X‐100. Then, Cells were incubated with primary rabbit antibody against PITPNM3 (1:100 dilution, Novus, Littleton, CO), E‐cadherin (1:100 dilution, Santa Cruz, CA), Vimentin and MTDH (1:100 dilution, Proteintech Group), and p65 (1:100 dilution, Cell Signaling Technology) at 4°C overnight. Cells were washed thrice with PBST and incubated with Alexa Fluor 594 goat anti‐rabbit IgG (H + L) (1:2000 dilution, Jackson Immuno Research, West Grove, PA) and Alexa Fluor 488 goat anti‐rabbit IgG (H + L) (1:500 dilution, Cell Signaling Technology) for 50 minutes in a dark humidified box and counterstained with 4’, 6‐diamidino‐2‐phenylindole. The images were captured using Leica DFC 450 fluorescence microscope (Leica Microsystems, Wetzlar, Germany).

### 2.8 Wound healing and Transwell invasion assay

For wound healing assay, Tu686, 6‐10B and FaDu cells were seeded in six‐well plates and allowed to reach 90% confluency. Cells were wounded by removing a line of cells with a disinfected micropipette tip (Eppendorf, Hamburg, Germany) and stimulated with rhCCL18 protein for 48 hours. Images of wounded areas were captured using a microscope. These experiments were performed in triplicates.

For Transwell invasion assay, Transwell chambers with polycarbonate filter were used, which were coated with 200 μg/mL of Matrigel (Corning, NY). Following incubation with rhCCL18 for 48 hours, transfected cells were seeded in Transwell cell culture inserts at a concentration of 2 × 10^4^ cells/well. The cells were then incubated at 37°C with 5% CO_2_ for 48 hours. The non‐invading cells on the upper side were removed using a cotton swab. Cells that had invaded to the lower surface were fixed with 10% buffered formalin, stained with crystal violet solution, photographed and counted.

### 2.9 Statistical analyses

GraphPad Prism (version 5.01, GraphPad Software, Version X; La Jolla, CA) and SPSS (version 22.0, SPSS Inc, Version X; IBM, Armonk, NY) were used for statistical analysis of the data. Unpaired *t*‐test, non‐parametric test or one‐way ANOVA test were performed to analyse the significant differences between groups. The data are expressed as mean ± standard deviation of mean. Chi‐squared test was used to analyse the relationship between CCL18 levels and clinicopathological features. *P* < 0.05 was considered statistically significant and two‐tailed tests were performed.

## 3 RESULTS

### 3.1 Serum CCL18 levels increased in SCCHN patients

**Figure 1:**
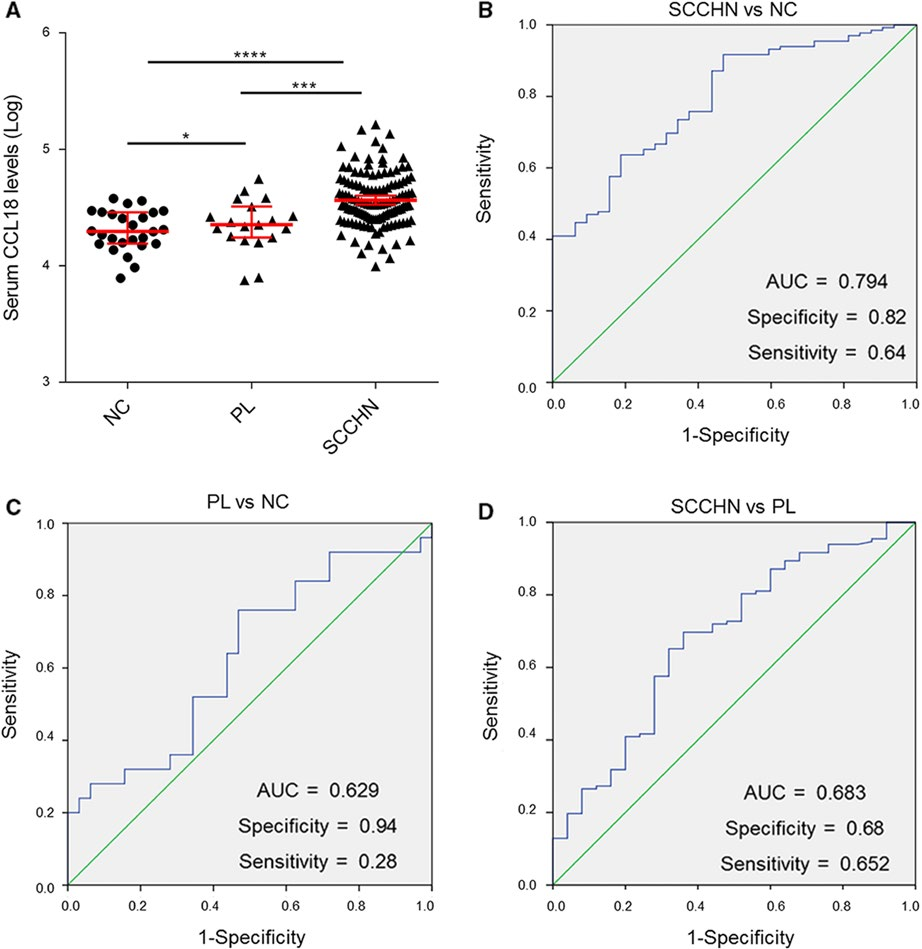
Serum level of CCL18 is significantly high in SCCHN patients. A, Levels of CCL18 in the serum of patients with SCCHN, precancerous lesions and healthy controls (*P < 0.05,***P < 0.001,****P < 0.0001 vs control). B‐D, ROC analysis showing the area under curve of serum CCL18 to distinguish (B) SCCHN patients from healthy controls, (C) precancerous lesions of SCCHN patients from healthy controls and (D) SCCHN patients from precancerous lesions of SCCHN. CCL18, chemokine (C‐C motif) ligand 18; NC, normal healthy controls; PL, precancerous lesions of SCCHN patients; ROC, receiver operating characteristic; SCCHN, squamous cell carcinoma of the head and neck

To confirm the relationship between CCL18 and SCCHN, blood samples were initially collected from SCCHN patients (n = 132), precancerous lesions (n = 25) and healthy donors (n = 32). Subsequently, ELISA results showed that the serum levels of CCL18 in SCCHN patients were 42134 ± 2245 pg/mL, while those of precancerous lesions of SCCHN patients and healthy donors were 28945 ± 2851 and 22551 ± 1544 pg/mL respectively (*P* < 0.0001) (Figure 1A). Moreover, 87.12% (115/132 samples) of the SCCHN samples had CCL18 levels higher than the maximum value measured in the healthy control samples. Levels of CCL18 in the serum from peripheral blood of SCCHN patients were significantly higher than the precancerous lesions of SCCHN patients and healthy controls, which suggested that serum CCL18 levels can serve as a potentially useful biomarker for the diagnosis and prognosis of SCCHN.

### 3.2 Correlation of serum CCL18 levels with clinicopathological parameters in SCCHN patients

Since serum CCL18 levels were elevated in SCCHN patients, we analysed the relationship between serum CCL18 levels and the major clinicopathological parameters in SCCHN patients (Table 1). As shown in Table 1, serum CCL18 levels were found to be significantly associated with tumour classification (T1 + T2 vs T3 + T4; *P* = 0.001), clinical stage (I + II vs III + IV; *P* = 0.000), lymph node metastasis (N0 vs N+; *P* = 0.001) and histological grade (Highly differentiated vs other differentiation stages, *P* = 0.021). However, serum CCL18 levels were not found to be correlated with age (*P* = 0.811), gender (*P* = 0.630), history of smoking (*P* = 0.757), alcohol consumption (*P* = 0.923) and primary tumour site (*P* = 0.271). Based on these results, serum CCL18 levels can be considered as biomarker for predicting the malignant progression and diagnosis of SCCHN.

### 3.3 Performance of serum CCL18 in diagnosis of SCCHN patients

To evaluate the performance of serum CCL18 in diagnosis of SCCHN patients, receiver operating characteristic (ROC) curve analysis was performed for SCCHN patients, precancerous lesions and healthy controls. Area under the ROC curve (AUC) for assessing the possibility of distinguishing SCCHN patients from healthy controls was 0.794 (specificity: 0.82, sensitivity: 0.64, cut‐off value: 29927.73 pg/mL, Figure 1B). AUC for evaluating the diagnostic accuracy of precancerous lesions from healthy controls was 0.629 (specificity: 0.94, sensitivity: 0.28, cut‐off value: 36897.26 pg/mL, Figure 1C). Furthermore, we also evaluated the diagnostic value for SCCHN from precancerous lesions, and the AUC was 0.683 (specificity: 0.68, sensitivity: 0.652, cut‐off value: 29015.51 ng/mL, Figure 1D). In conclusion, a cut‐off value of 29927.73 pg/mL for serum CCL18 (SCCHN vs. healthy controls) was applied for further analysis and clinical diagnosis.

### 3.4 CCL18 promotes metastasis and EMT in SCCHN cells

**Figure 2:**
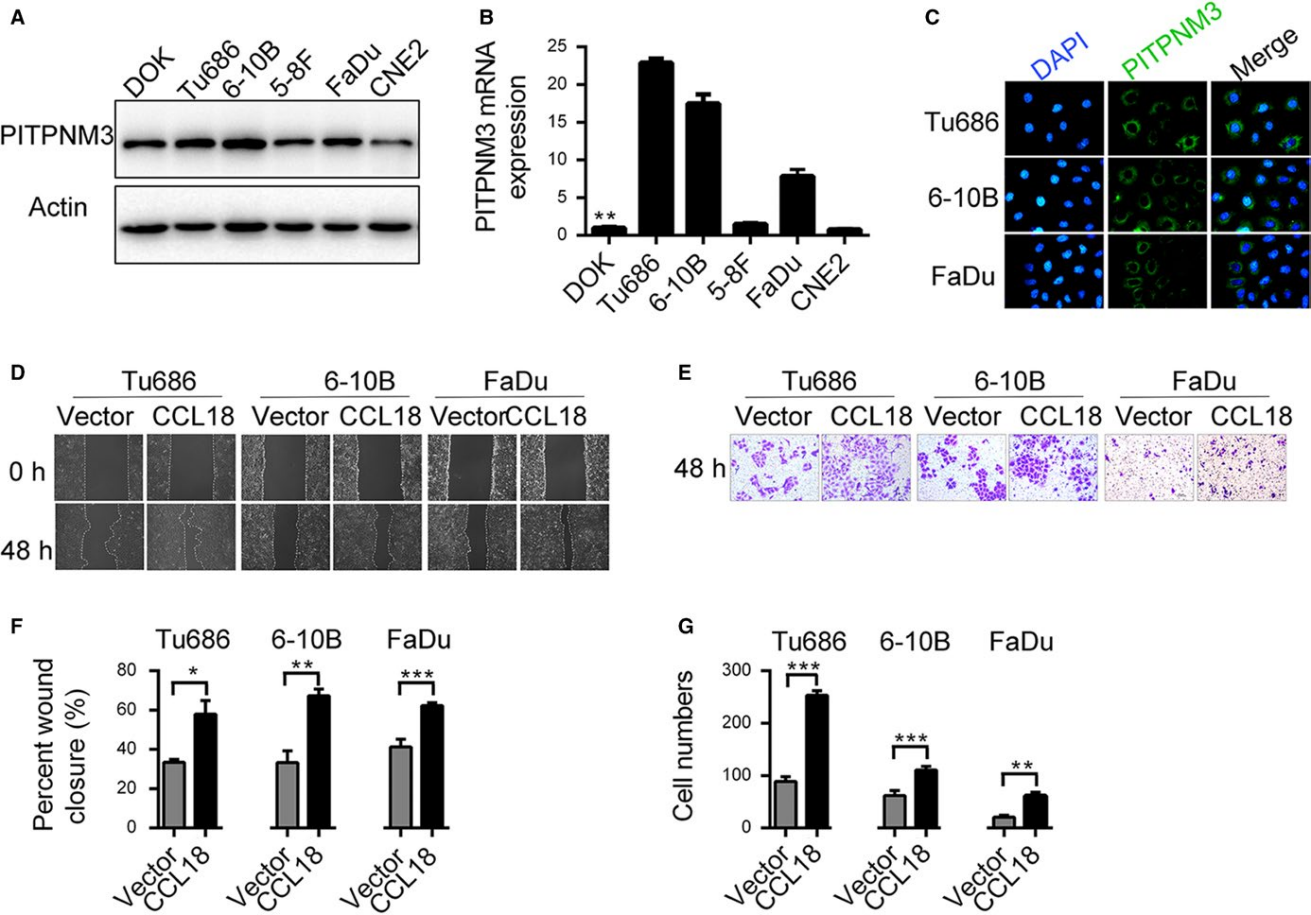
CCL18 promotes migration and invasion of SCCHN cells in vitro. Relative expression level of PITPNM3 protein in (A) five different SCCHN cell lines and (B) an immortalized non‐malignant cell line derived from oral mucosa (DOK). C, Expression of PITPNM3 protein in SCCHN cell lines. D‐G, Effect of rhCCL18 on migration (D and F) and invasion (E and G) of SCCHN cells. (*P < 0.05, **P < 0.01, ***P < 0.001 vs vector). CCL18, chemokine (C‐C motif) ligand 18; DOK, dysplastic oral keratinocyte; rhCCL18, recombinant human CCL18; SCCHN, squamous cell carcinoma of the head and neck

Several studies have identified that CCL18 specifically binds to PITPNM3 on the cellular membrane to exert its biological effects. Thus, we initially detected the expression of PITPNM3 in different cell lines used in this study. Tu686, 6‐10B and FaDu exhibited high levels of PITPNM3 expression, both at mRNA and protein levels, compared to the DOK cells (Figure 2A‐C).

Treatment with rhCCL18 for 24, 48, 72 and 96 hours did not have any significant effect on cell proliferation and differentiation in Tu686, 6‐10B and FaDu cells (Figure S1B). Similar results were obtained in plate cloning experiments (Figure S1C). Wound healing assay demonstrated that cells treated with rhCCL18 migrated more quickly than the control untreated cells (Tu686 cells: 33.38 ± 0.91% vs 57.97 ± 3.98%, respectively, *P* < 0.05; 6‐10B cells: 33.31 ± 3.44% vs 67.35 ± 1.97%, respectively, *P* < 0.01; FaDu cells 41.23 ± 2.28% vs 62.34 ± 0.80%, respectively, *P* < 0.001, Figure 2D and F). Similar results were also observed in the Transwell invasion assays (Tu686 cells: 89 ± 4 vs 254 ± 4, respectively, *P* < 0.0001, 6‐10B cells: 62 ± 4 vs 111 ± 3, *P* < 0.0001, FaDu cells: 21 ± 2 vs 61 ± 3, *P* < 0.0001, Figure 2E and G). These results suggest that CCL18 plays an important role in promoting cell migration and invasion of SCCHN cells in vitro.

**Figure 3:**
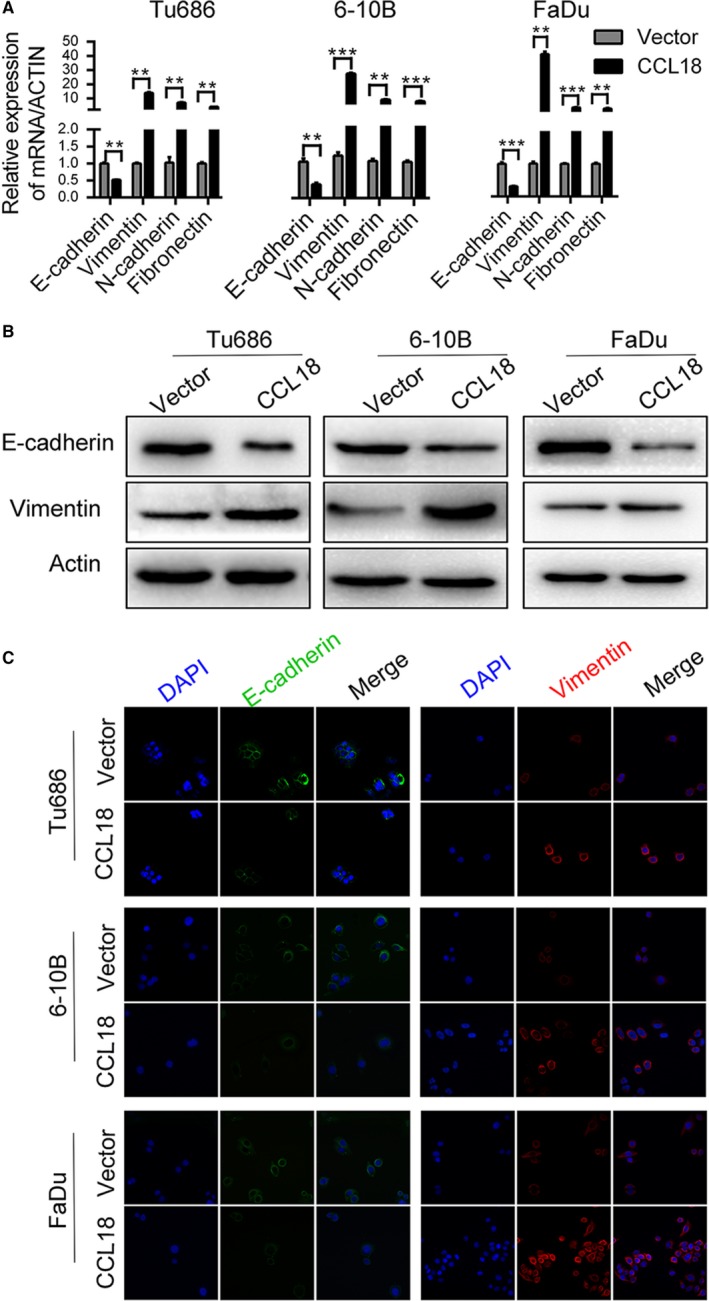
rhCCL18 promotes EMT in SCCHN cells. A, Effect of rhCCL18 on EMT markers at mRNA and (B and C) protein level (**P < 0.01, ***P < 0.001 vs vector). EMT, epithelial‐mesenchymal transition; rhCCL18, recombinant human CCL18; SCCHN, squamous cell carcinoma of the head and neck

Furthermore, EMT is closely related to cancer metastasis, which can typically be characterized by different molecular markers.^38^, ^39^ In this study, treatment with rhCCL18 down‐regulated the expression of E‐cadherin, an epithelial cell marker, and up‐regulated expression of mesenchymal markers such as Vimentin, N‐cadherin and Fibronectin at mRNA level (Figure 3A). Similarly, treatment with rhCCL18 protein decreased the expression of E‐cadherin and increased the expression of Vimentin at protein level as assessed by Western blot and immunofluorescence experiments (Figure 3B and C).

### 3.5 MTDH is essential for CCL18‐induced migration, invasion and EMT in SCCHN cells

**Figure 4:**
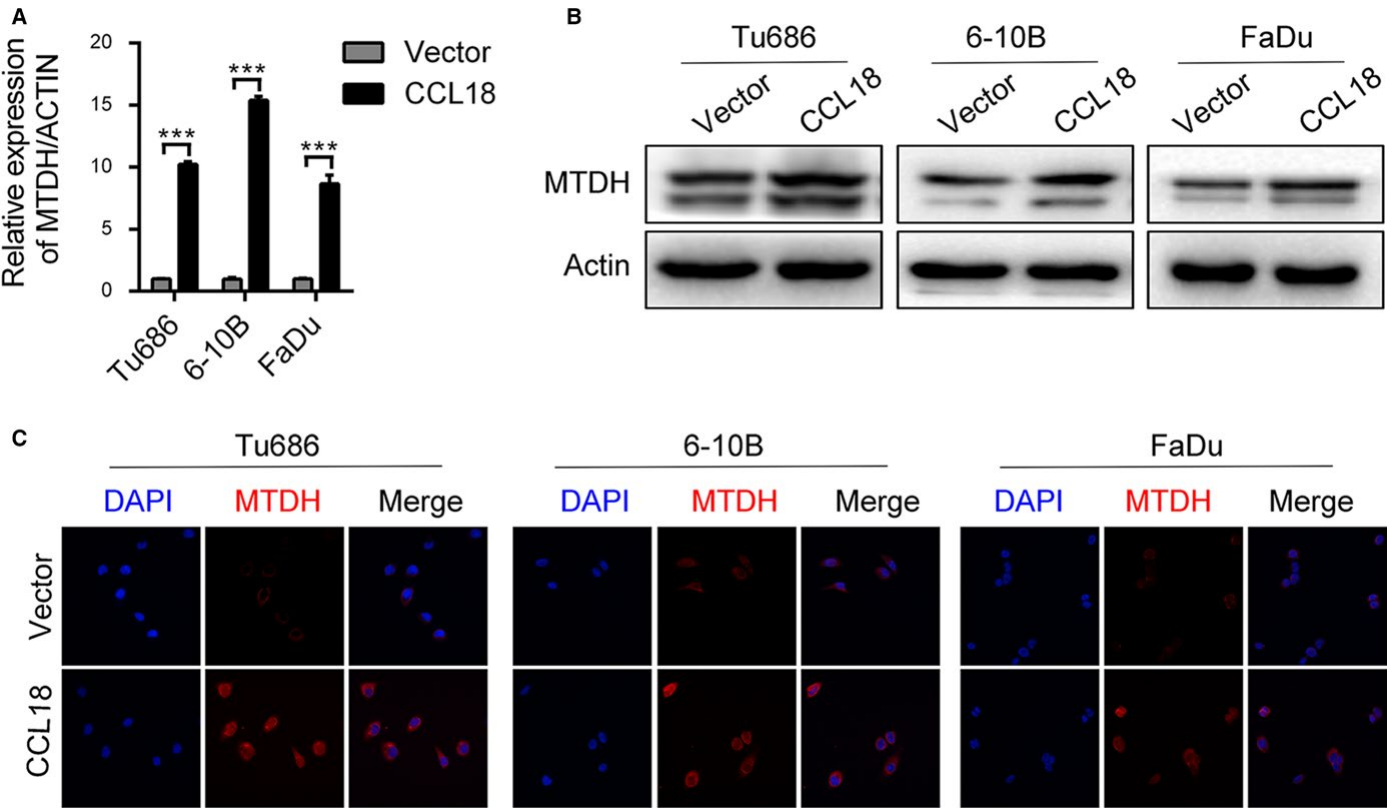
rhCCL18 promotes the expression of MTDH. A, Effect of CCL18 on the expression of MTDH at mRNA and (B and C) protein level (***P < 0.001 vs vector). CCL18, chemokine (C‐C motif) ligand 18; MTDH, metadherin; rhCCL18, recombinant human CCL18

In our previous study, we found that MTDH plays a very important role in the invasion, metastasis and EMT of SCCHN cells.^38^, ^39^ To identify a potential target molecule of CCL18 which may mediate its biological effects, we examined the mRNA and protein expression of MTDH in rhCCL18‐treated Tu686, 6‐10B and FaDu cells. Our results showed that treatment with rhCCL18 for 48 hours increased the mRNA levels of MTDH in all three cell lines (Figure 4A). Western blot and immunofluorescence analysis also revealed an increase in the protein levels of MTDH (Figure 4B and C), which was consistent with an increase in its mRNA levels.

To further confirm the effect of MTDH on rhCCL18‐mediated invasion and metastasis of SCCHN cells in vitro, we developed stable transgenic Tu686, 6‐10B and FaDu cell lines with knockdown of MTDH (Figure S2A).

**Figure 5:**
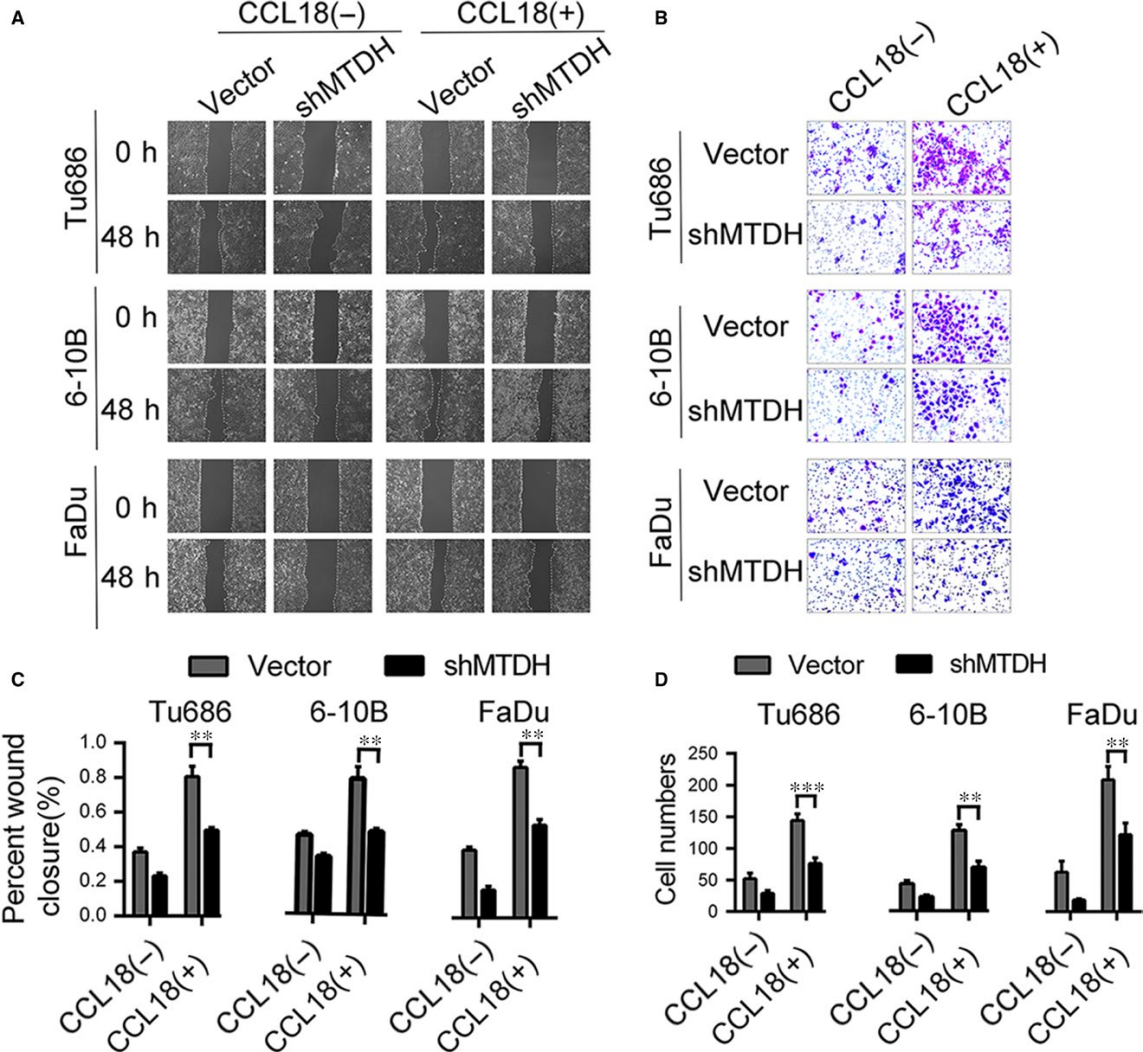
rhCCL18 promotes migration and invasion of SCCHN cells in an MTDH‐dependent manner. A and C, Migratory changes were examined and quantified by wound healing assay. B and D, Invasive ability was assayed and quantified by Transwell invasion assay. (**P < 0.01, ***P < 0.001) MTDH, metadherin; rhCCL18, recombinant human CCL18; SCCHN, squamous cell carcinoma of the head and neck

Wound healing assay was performed to evaluate the effect of rhCCL18 on migration in MTDH‐knockdown Tu686, 6‐10B and FaDu cells. As shown in Figure 5A, treatment with rhCCL18 increased the invasion of Tu686, 6‐10B and FaDu cells, and this was significantly reduced in MTDH‐knockdown SCCHN cells (*P* < 0.001). Similar results were obtained by the Transwell invasion assay where treatment with rhCCL18 significantly enhanced the migration and invasion of Tu686, 6‐10B and FaDu cells. This effect was significantly attenuated in MTDH knockdown cells (*P* < 0.001, Figure 5B and D). Thus, these results suggest that MTDH is involved in promoting metastasis of SCCHN cells via expression of CCL18.

**Figure 6:**
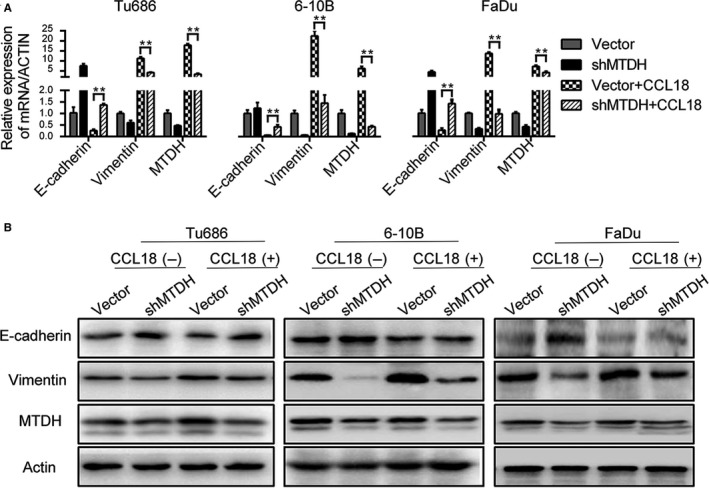
Knockdown of MTDH impaired the rhCCL18‐induced EMT in SCCHN cells. A, mRNA levels and (B) protein levels. (**P < 0.01) EMT, epithelial‐mesenchymal transition; MTDH, metadherin; rhCCL18, recombinant human CCL18; SCCHN, squamous cell carcinoma of the head and neck

Additionally, we analysed molecular markers of EMT by qRT‐PCR and Western blot in MTDH knockdown cells. Treatment with rhCCL18 for 48 hours did not increase the expression of Vimentin at both mRNA and protein level in MTDH knockdown cells. Down‐regulation of E‐cadherin showed no obvious trend in SCCHN cells at both the mRNA (Figure 6A) and protein levels (Figure 6B). These results suggest that the biological effect of CCL18 in promoting EMT in SCCHN cells is dependent on the expression of MTDH.

### 3.6 NF‐κB activation is involved in CCL18/MTDH‐mediated metastasis and EMT of SCCHN cells

**Figure 7:**
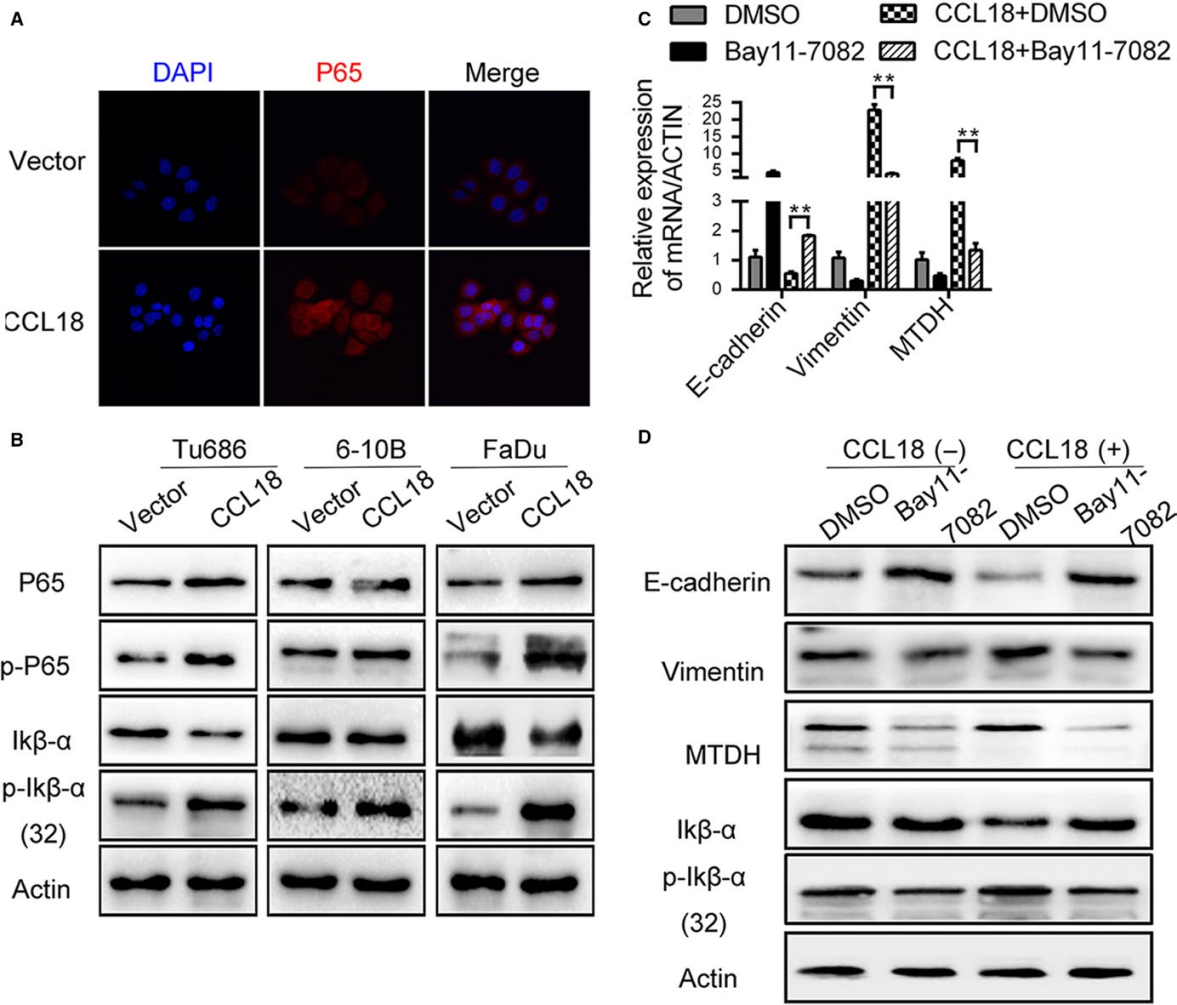
NF‐κB activation is essential for CCL18/MTDH‐mediated cell migration, invasion and EMT in SCCHN cells. A, Representative immunofluorescence images show that CCL18 induces nuclear translocation of p65. B, Expression of NF‐κB‐associated protein subsequent to rhCCL18 stimulation. C and D, rhCCL18 enhanced the EMT property featured by E‐cadherin and Vimentin expression (**P < 0.01). This effect of CCL18 is reduced by repressing NF‐κB activation in Tu686 cells. (C) mRNA levels and (D) protein levels. CCL18, chemokine (C‐C motif) ligand 18; EMT, epithelial‐mesenchymal transition; MTDH, metadherin; rhCCL18, recombinant human CCL18; SCCHN, squamous cell carcinoma of the head and neck

To further investigate the mechanism by which CCL18/MTDH mediates migration, invasion and EMT in SCCHN cells, we measured the phosphorylation status of p65 and IκB‐α using Western blotting. As shown in Figure 7B, treatment with rhCCL18 significantly increased the expression of p65 and also increased the phosphorylation of p65 and IκB‐α in Tu686, 6‐10B and FaDu cells. We also observed nuclear translocation of p65 using immunofluorescence staining method (Figure 7A). Interestingly, rhCCL18‐mediated increase in the expression of IκB‐α and p‐IκB‐α was significantly attenuated in presence of Bay11‐7082, an inhibitor of phosphorylation of IκB‐α. These results implied that activation of NF‐κB is required for CCL18/MTDH‐mediated metastasis and EMT in SCCHN cells.

Expression of MTDH and EMT molecular markers such as E‐cadherin and Vimentin at both mRNA and protein level was measured in Tu686 cells, after treated with 5 μmol/mL of Bay11‐7082. The results showed that treatment with rhCCL18 significantly increased the expression of Vimentin and MTDH, while decreased the expression of E‐cadherin in Tu686 cells. As expected, treatment with Bay11‐7082, a specific inhibitor of phosphorylation of IκB‐α, inhibited this effect of rhCCL18 in inducing EMT, as evidenced by the expression of E‐cadherin, Vimentin and MTDH which were found to be restored (Figure 7D).

## 4 DISCUSSION

CCL18, a CC chemokine produced by M2 macrophages, is known to correlate with cancer initiation and progression. In the present study, serum CCL18 levels were found to be significantly high in patients with SCCHN compared to precancerous lesions of SCCHN patients and healthy controls, and its levels correlated with tumour classification, clinical stage, lymph node metastasis and histological grade in SCCHN patients. Most importantly, we observed that treatment with rhCCL18 protein promoted the migration and invasion of SCCHN cells in vitro. Furthermore, MTDH played a pivotal role in mediating the biological effect of CCL18, and knockdown of MTDH impaired the CCL18‐induced migration, invasion and EMT in SCCHN cells. We also found a crosstalk mechanism between CCL18/MTDH and EMT in SCCHN cells. Briefly, treatment with rhCCL18 enhanced the expression of MTDH, which was accompanied by transcriptional activation of NF‐κB resulting from phosphorylation of IκB‐α as well as nuclear translocation of p65.

The tumour microenvironment consists of a variety of non‐malignant stromal cells that play a key role in tumour progression and metastasis.^48^ Among them, TAMs are the most notable type of migratory hematopoietic cells.^49^, ^50^ Many studies including our previous findings demonstrated that CCL18 is a vital chemokine produced mainly by TAMs and it is associated with progression of various malignant tumours.^9^, ^10^, ^11^, ^14^, ^15^, ^17^, ^18^, ^22^, ^25^ Moreover, therapeutic success in inhibiting these tumorigenic activities in preclinical models and in early clinical trials suggests that macrophages are attractive targets as a part of the combination therapy in cancer treatment.^48^ Our ELISA results revealed that serum levels of CCL18 were significantly high in patients with SCCHN compared to the precancerous lesions of SCCHN patients and healthy controls. Furthermore, serum CCL18 levels correlated with tumour classification, clinical stage, lymph node metastasis and histological grade significantly. These findings are consistent with several previous studies on breast cancer, non‐small cell lung cancer, ovarian cancer and pancreatic ductal adenocarcinoma, where high levels of CCL18 in blood has been reported to be associated with an increased occurrence of metastasis and tumour invasion, which results in poor prognosis.^10^, ^22^, ^36^, ^51^ Cancer metastasis begins with cancer cell migration or invasive growth, which then penetrates through nearby lymphatic or vascular wall. Consistent with our ELISA results, we observed that rhCCL18 protein promoted the migration and invasion of SCCHN cells in vitro. EMT has been demonstrated to play a pivotal role in the process of cell migration and invasion in a variety of cancer types including SCCHN.^37^, ^45^, ^52^ Cancer cells that undergo EMT are characterized by down‐regulation of cell‐cell adhesion molecules, such as E‐cadherin, and up‐regulation of Vimentin and N‐cadherin.^37^

Recent studies have demonstrated that CCL18 specifically binds to PITPNM3 on the cellular membrane of breast cancer cells and hepatocellular carcinoma to exert its biological effects.^9^, ^10^, ^11^, ^12^, ^13^, ^14^ CCL18 also acts on a variety of target molecules, including VCAM‐1, AMAP1 and ACAP4.^24^, ^40^, ^46^ In our study, SCCHN cells with high PITPNM3 expression were chosen for transfection with rhCCL18. Interestingly, MTDH expression increased at both mRNA and protein levels in rhCCL18‐treated SCCHN cells Furthermore, stable transfection with shMTDH reversed the effect of CCL18 on SCCHN cells as shown in the wound healing and Transwell invasion assay. Our data demonstrated that MTDH may serve as an essential target of CCL18 in SCCHN cells.

Although our results confirmed that MTDH plays a pivotal role in inducing biological effects of CCL18, the mechanism by which CCL18 up‐regulates the expression of MTDH is not clear. Previous studies demonstrated that CCL18 plays an important role in migration, invasion and EMT of cancer cells via the PI3K‐AKT signalling pathway.^53^, ^54^ Furthermore, it has been shown that H‐ras markedly induced expression of MTDH through PI3K‐AKT signalling pathway augmenting binding of c‐Myc to E‐box elements in the promoter region of MTDH gene, thereby regulating its transcription.^30^ Since CCL18 activates the PI3K‐AKT pathway, and AKT in turn promotes MTDH transcription, it is interesting to study whether CCL18 up‐regulates the expression of MTDH by binding to PITPNM3 to activate the PI3K‐AKT pathway.

Previous studies have reported that production of CCL18 by TAMs induced NF‐κB activation in hepatocellular carcinoma metastasis.^14^ This is in agreement with our results showing that NF‐κB nuclear translocation and the classic signalling pathway was activated in CCL18‐stimulated SCCHN cells. However, the mechanism by which expression of NF‐κB is regulated is still not known. The present study demonstrated that NF‐κB signalling was mediated by MTDH in CCL18‐stimulated SCCHN cells. MTDH regulates multiple signalling pathways, including NF‐κB signalling. MTDH has been shown to facilitate the translocation of NF‐κB into the nucleus and interacts with the p65 subunit of NF‐κB which acts as a coactivator for NF‐κB to modulate gene expression of targeted genes.^45^, ^55^ Moreover, IκB kinase β‐mediated MTDH phosphorylation is critical for IκB‐α degradation as well as NF‐kB‐dependent gene expression and cell proliferation, which correlates with survival in cancer patients.^56^

Thus, to summarize, our study demonstrated that serum levels of CCL18 may serve as potential biomarker for diagnosis of SCCHN, and CCL18 plays an important role during invasion, metastasis and EMT of SCCHN. Moreover, MTDH is at the core of CCL18‐induced biological effects. Activation of NF‐κB is involved in CCL18/MTDH‐induced EMT, which intrinsically enhances the ability of cell migration and invasion, suggesting that MTDH may be used as a therapeutic target for the inhibition of SCCHN metastasis.

## Supporting information

FigS1

FigS2
