# Supplementary figures and images for: CCL18 promotes the metastasis of squamous cell carcinoma of the head and neck through MTDH‐NF‐κB signalling pathway
Source: J Cell Mol Med. 2019 Feb 15;23(4):2689–701. doi: 10.1111/jcmm.14168 (PMC6433669; doi:10.1111/jcmm.14168)

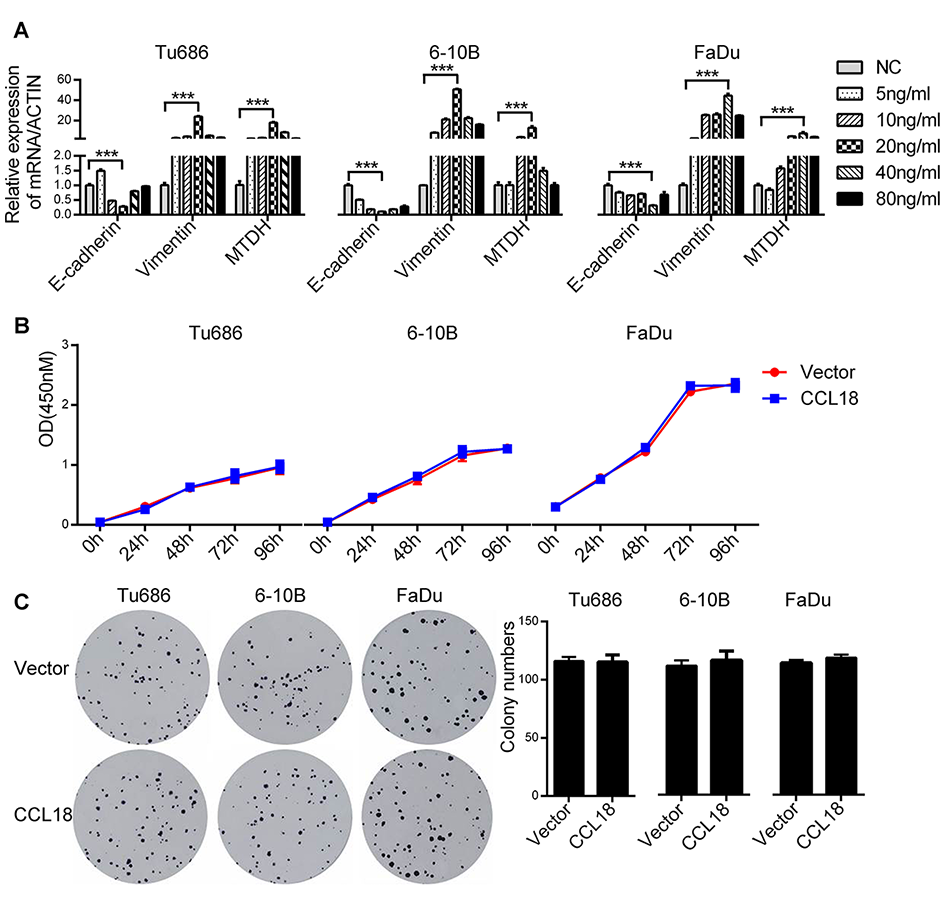

Supplement: Supplementary file 1 — FigS1 [file JCMM-23-2689-s001.tif]

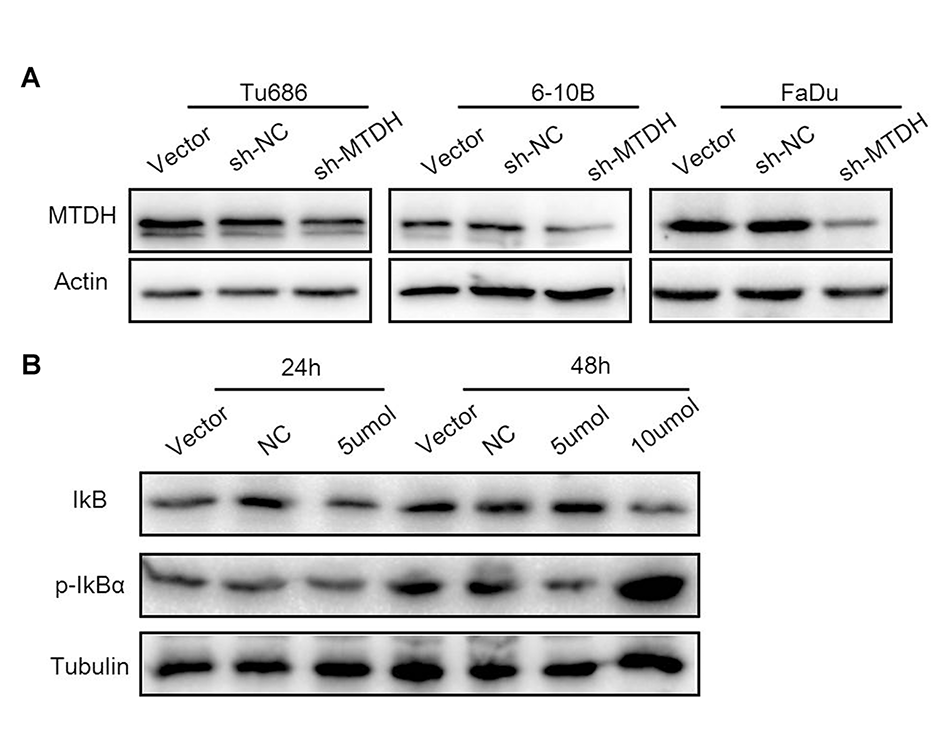

Supplement: Supplementary file 2 — FigS2 [file JCMM-23-2689-s002.tif]
